# Hydroxycamptothecin regulates scar formation of the filtration channel under scleral flap by inhibiting the proliferation of scleral fibroblasts

**DOI:** 10.1371/journal.pone.0284618

**Published:** 2023-04-20

**Authors:** Hongwei Gu, Ya Liang, Yao Shen, Jie Shuai, Qiuli Yu, Huaijin Guan, Zhilan Yuan

**Affiliations:** 1 Department of Ophthalmology, The First Affiliated Hospital of Nanjing Medical University, Nanjing, Jiangsu Province, China; 2 Department of Ophthalmology, Affiliated Hospital of Nantong University, Nantong, Jiangsu Province, China; 3 Department of Ophthalmology, Nanjing Medical University, The Second Clinical Medical College of Nanjing Medical University, Nanjing, Jiangsu Province, China; University of Missouri-Columbia, UNITED STATES

## Abstract

**Background:**

To investigate the inhibitory effect of a hyaluronic acid hydrogel loaded with hydroxycamptothecin (HCPT) on scar formation after filtration surgery in a rabbit model.

**Methods:**

Scleral fibroblasts were isolated and extracted from rabbits’ eyes. After treatment with different concentrations of HCPT, cytotoxicity was detected using the 3-(4,5-dimethylthiazol-2-yl)-2,5-diphenyltetrazolium bromide assay, and proliferation and extent of apoptosis were analysed using flow cytometry. Hydrogels loaded with different dosages of HCPT were prepared and placed under the scleral flap after the filtration surgery. One day, one week, and two weeks after surgery, follicular, conjunctival, corneal, and anterior chamber inflammation and iris and lens changes were observed.

**Results:**

In vitro, compared with cells not treated with HCPT, cells treated with HCPT had decreased survival rate and proliferation, and the apoptosis level increased with increasing HCPT concentrations (p < 0.05). In vivo, the flattening time of filtering blebs in the three groups treated with different dosages of HCPT hydrogel was delayed. The degrees of oedema, inflammation, and bleeding were similar to those observed in the control group. The HCPT hydrogel effectively downregulated the expression of collagen 1 and 3 and tissue inhibitor of metalloproteinase 2 and upregulated the expression of matrix metalloproteinase 2 in a dose-dependent manner.

**Conclusions:**

HCPT significantly inhibited the growth of rabbits’ scleral fibroblasts and effectively inhibited scar formation after filtering surgery by accelerating the degradation of extracellular matrix deposition.

## Introduction

Approximately 60.5 million people worldwide have primary glaucoma. The prevalence of glaucoma in individuals aged 40–80 years is 3.54%. In 2013, 64.3 million people were diagnosed with glaucoma. The number of affected individuals is estimated to reach 76 million by 2020 and 111.8 million by 2040 [1]. Treatments for glaucoma include medications, lasers, and surgery, applied alone or in combination. Surgery is the best option for patients with glaucoma showing poor results with medication or laser treatment [2].

For glaucoma filtration surgery, classical trabeculectomy, mucosal tubular surgery, or drainage implantation remains the most common and effective treatment for lowering the intraocular pressure (IOP). The goal of these surgeries is to create a filtration channel for external drainage of atrial fluid from the anterior chamber through the scleral flap down to the subconjunctiva. Glaucoma trabeculectomy has a failure rate of up to 30% within 2 years. There are multiple causes of failure, with excessive subconjunctival and subscleral fibrosis in the operative area being the main cause [3]. In particular, proliferation of fibroblasts under the scleral flap narrows or even closes the external drainage filtration channel. Therefore, the combination of intraoperative and postoperative anti-fibrotic and anti-scarring drugs under the scleral flap during filtration surgery can help suppress the excessive healing response in the operative area, control the target IOP, and maintain stable IOP in the long term.

Hydroxycamptothecin (HCPT) is an endostatin alkaloid and a hydroxy derivative of camptothecin. HCPT targets the cell cycle and acts in the S phase of cells. It has fewer toxic side effects and high and broad anti-tumor activity and is commonly used clinically for the treatment of gastric, bladder, and colorectal cancers [4,5]. In contrast, mitomycin-C (MMC) and 5-fluorouracil (5-FU) are cell cycle non-specific antimetabolites that have significant blocking effects on all cell cycle phases.

HCPT is being increasingly used in the eye to study its role as immunosuppression and anti-fibrotic agents [6]. In this study, a slow-release hyaluronic acid hydrogel containing HCPT was applied to the scleral filtration channel of rabbit glaucoma to achieve an effective, non-toxic, and sustained inhibition of filtration vesicle scar formation. Our findings will provide a new technique and method for inhibiting filtration channel scar formation following glaucoma filtration surgery.

## Materials and methods

### Cell cycle analysis

Cells were seeded into six-well plates at a density of 1.5 × 10^5^ cells/well. The drugs were added to the plates and incubated in a CO_2_ incubator for a defined period. The cells were collected in a centrifuge tube and centrifuged at 1,000 rpm for 5 min. The supernatant was almost completely discarded, and the cells that remained in the remaining approximately 50 μL were resuspended in 2 mL of added phosphate buffered saline (PBS). Then, 1 mL of pre-cooled 70% ethanol was slowly added to the cell suspension, and the centrifuge tube was shaken to reduce cell aggregation. The cells were fixed in ethanol overnight at 4°C. Two millilitres of PBS were added to the fixed cells. The mixture was centrifuged at 1,000 rpm for 5 min, and the supernatant was discarded. The washing steps described above were then repeated. The final cell pellet was resuspended in 0.5 mL PBS. Ribonuclease and propidium iodide (PI) were added. The sample was examined using flow cytometry (Attune NxT, Invitrogen) with excitation and scattered light wavelengths of 488 and 585 nm, respectively [7].

### Cell viability assay

Cells were inoculated into 96-well plates at a density of 0.5×10^4^ cells/well. After 24 hours, the cells were washed twice with PBS. In the final pellet, 200 μL of nanoparticle suspensions of different concentrations diluted in serum-free Dulbecco’s modified Eagle’s medium (DMEM) were added. Different concentrations of nanoparticles were used, with one concentration per sample. Transfected liposomes were used as positive controls. Blank and negative control wells were also included. Each treatment was performed twice for each group, with triplicates being performed per treatment. After 24 h of incubation, 20 μL of a 5 mg/mL solution of 3-(4,5-dimethylthiazol-2-yl)-2,5-diphenyltetrazolium bromide (MTT; pH 7. 4) was added to each well, and incubation was continued for 4 h. Then, 150 μL of dimethyl sulfoxide (DMSO) was added to each well and shaken for 10 min to fully dissolve the crystals. The absorbance was measured at 450 nm using a microplate reader (Bio-Rad, Hercules, CA, USA) [8–10].

### Cell apoptosis assay

To prepare hyaluronic acid (HA) with an alkyne group in the side chain, 200 mg of HA was dissolved in 15 mL of MES buffer solution (50 mM, pH 4.0) with constant stirring to dissolve it completely. This was followed by the addition of 478 mg (2.49 mmol) 1-ethyl-3-(3-dimethylaminopropyl)carbodiimide (EDC), 287 mg (2.5 mmol) N-hydroxysuccinimide, and 195.1 mg (3.47 mmol) propargylamine (PA), with constant stirring at room temperature for 24 h. At the end of the reaction, the product was dialysed in a dialysis bag in saturated sodium chloride solution for 24 h, followed by dialysis in distilled water for 5 days. The modified alkyne-based HA was lyophilised. HA hydrogels were prepared by the click reaction using azide-polyethylene glycol-azide as a cross-linking agent and sodium ascorbate and copper sulfate as catalysts. HCPT was added, and solidified pharmaceutical hydrogels were obtained as previously described [11,12].

### Preparation of HCPT HA hydrogel

HA hydrogels containing different concentrations of HCPT (0.125, 0.25, and 0.5 mg/mL) were configured and partially dried at 37°C to form a film. A representative 4×4 mm piece of a dry film was further examined using electron microscopy.

### Real-time quantitative polymerase chain reaction (RT-qPCR)

A 25 μL solution was prepared containing the following ingredients: complementary DNA 2 μL, 2×SYBR Green Master Mix Buffer 12.5 μL, upstream primer (10 mol/L) 1 μL, downstream primer (10 mol/L) 1 μL, and distilled deionised water 8.5 μL. The solution was gently mixed, centrifuged, and amplified by PCR using the ABI7500 real-time PCR system (Thermo Fisher Scientific). The conditions for each cycle of PCR amplification were as follows: pre-denaturation at 95°C for 2 min, denaturation at 95°C for 10 s, annealing at 60°C for 30 s, and extension at 72°C for 30 s. This procedure consisted of 40 cycles. Three replicates were performed for each sample, and glyceraldehyde 3-phosphate dehydrogenase (GAPDH) was used as the internal control. Cycle threshold (Ct) values were determined and statistically analysed, as previously described [13–15].

### Western blotting analysis

Proteins were separated using 10% sodium dodecyl sulfate (SDS)-polyacrylamide gel electrophoresis (10 μL per well) at 90 V for 20 min for the concentrating gel and 120 V for 60 min for the separation gel. The resolved proteins were transferred to polyvinylidene fluoride (PVDF) membranes using the wet electrotransfer method with a constant current of 200 mA for 150 min. The membranes were washed twice with Tris-buffered saline-Tween (TBST; 50 mM Tris-HCl, 100 mM NaCl, 0.1% Tween-20, pH 7.6), blocked with 5% skim milk powder in TBST for 1 h at 25°C, and kept at 4°C until analysis. Then the membranes were incubated in primary antibodies for target proteins and the loading control protein (Col I, Col III, FN, MMP2, TIMP2, Decorin, and GAPDH) overnight under ambient conditions, followed by TBST washing of the membrane three times for 5 min each time. Afterwards, the membranes were incubated in secondary antibodies overnight at 4°C. Finally, the membranes were washed thrice (5 min each time) with TBST. The PVDF membranes were scanned using a molecular odyssey infrared imaging system (Li-COR Biosciences), and the bands were analysed. Integrated optical density was used to determine the relative protein content, as previously described [16–18].

#### Culture of rabbits’ scleral fibroblasts

The rabbits’ eyeballs were removed, and the bulbar conjunctiva, fascia, muscle tissue, retina, and choroid were retained. The posterior sclera were cut into tissue blocks of approximately 1×1 mm and evenly spread in dishes containing complete medium (90% DMEM and 10% foetal bovine serum supplemented with 100 mg/mL streptomycin and 100 U/mL penicillin). The dishes were incubated at 37°C for 7–14 days to allow the separation of the cells from the tissue blocks and cell adhesion, fusion, and proliferation. When the cells were 90% confluent, they were washed twice with PBS, and trypsin was added to detach the cells. The collected cells were centrifuged, the supernatant was discarded, and the cell pellet was resuspended in the culture medium. The cells were then cultured in cell culture flasks.

#### Selection and grouping of experimental animals

Fifteen sterile, 12-month-old New Zealand white rabbits with an average weight of 2.0 kg (both males and females) were provided by the Experimental Animal Center of Nantong University. The animals used in this experiment and experimental conditions were handled in accordance with the relevant regulations of the Regulations for the Administration of Laboratory Animals. The animal rearing room and clean environment complied with the standard conditions. The 15 rabbits were randomly divided into five groups (n = 3 per group): control group, gel group, 0.125 mg/mL HCPT group, 0.25 mg/mL HCPT group, and 0.5 mg/mL HCPT group. Routine preoperative ocular examinations were performed using slit-lamp microscopy, indirect fundoscopy, and other equipment to exclude ocular diseases. Levofloxacin eye drops were administered to the right eye three times daily for 3 days before surgery. After surgery, aureomycin ointment was applied to the right eye once a night.

#### Establishment of the rabbit model of sclera flap

The control group did not receive any medication under the scleral flap during the procedure. In the other groups, 4×4-mm squares of HCPT-free gel or gels with various concentrations of HCPT were placed under the scleral flap. The scleral flap was closed with three stitches using a 10–0 suture. Subsequently, the conjunctival flap was sutured. After the procedure, aureomycin ointment was applied to the conjunctival sac and levofloxacin ophthalmic solution was administered three times daily. The morphology and duration of the presence of filtering ophthalmia were observed and recorded using slit-lamp microscopy on postoperative days 1, 7, and 14. Slit-lamp microscopy and fundoscopy on the same postoperative days were used to detect complications including punctate detachment of the corneal epithelium, corneal oedema, conjunctival wound leakage, thin-walled follicles, anterior chamber swallowing, anterior chamber inflammation, anterior chamber haemorrhage, lens clouding, and fundus damage.

Each rabbit was anaesthetised with 2% sodium pentobarbital (1 mL/kg) and immobilised on an operating table. The surface of the right eye was anaesthetised using proparacaine hydrochloride eye drops. Routine disinfection was performed, and the sterile tissue was removed. The eyelid was opened, and the conjunctival sac was flushed with saline. A conjunctival flap was created from the superior temporal area based on the fornix. A scleral flap was created based on the corneoscleral rim. The rectangular flap was approximately 4×5 mm in size. The flap approximately half the scleral thickness was created starting from the posterior edge of the flap and incised to 1 mm inside the corneal rim. The trabecular tissues were excised. Trabecular tissue (1×2 mm) was excised under the scleral flap. An anterior incision was made at the clear edge of the cornea. At the time of sacrifice, animals were euthanised by CO_2_ inhalation.

### Protein extraction

The tissue was washed thrice with PBS. An appropriate amount of protein lysate containing a protease inhibitor was added (300 μL in each well of a six-well plate) and placed on ice for 30 min. The lysate was collected and centrifuged at 12,000 g for 20 min at 4°C. The supernatant was carefully aspirated, and the protein concentration was quantified using bicinchoninic acid assay. One-third volume of 4× loading buffer was added and mixed well, and the tube was placed in boiling water for 5 min. After cooling, the sample was divided into several aliquots and stored at −20°C for backup.

### Statistical analysis

The results are presented as mean ± standard deviation. SPSS 21 statistical software (IBM, Armonk, NY, USA) was used to perform the statistical analysis using Dunnett’s t-test for one-way analysis of variance. p < 0.05 was considered statistically significant.

## Results

### Hydroxycamptothecin inhibits the growth of rabbits’ scleral fibroblasts

**Fig 1:**
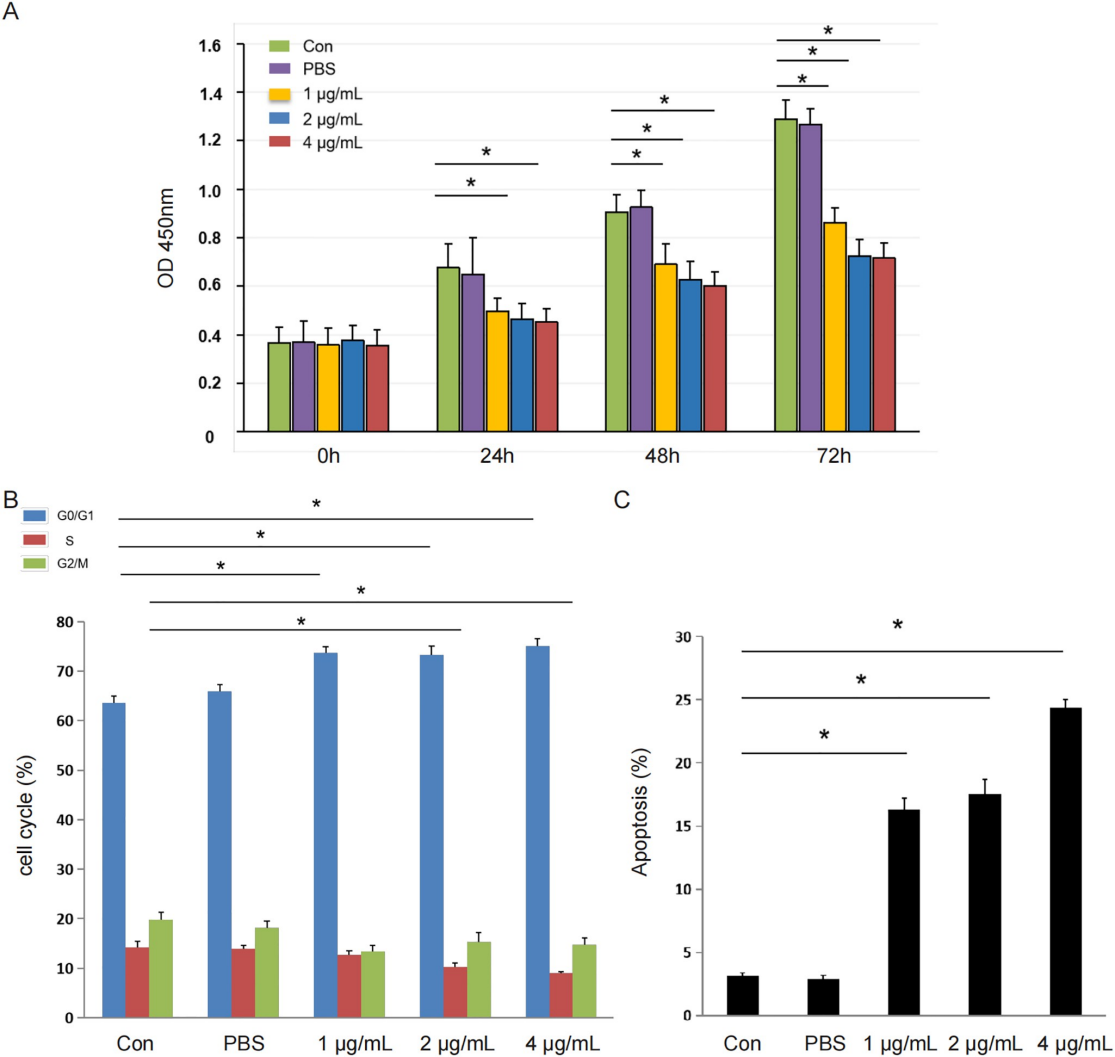
HCPT treatment effect. (A) MTT analysis was performed to evaluate the effect of different concentrations of HCPT on cell viability. HCPT had a statistically significant inhibitory effect on the growth of rabbits’ scleral fibroblasts in a concentration-dependent manner. (B) Cell cycle distribution of scleral fibroblasts examined by flow cytometry revealed a significant G0/G1 phase arrest after 24-h treatment of cells with different concentrations of HCPT, indicating the accumulation of cells in the G1 phase. S phase cells were decreased after treatment with HCPT at concentrations of 2 μg/mL and greater. (C) Annexin V and PI double staining to detect apoptosis after HCPT treatment at different concentrations revealed that HCPT significantly induced the development of scleral fibroblast apoptosis in a dose-dependent manner.

Rat scleral fibroblasts were treated with 1, 2, or 4 μg/mL HCPT. Cell survival was assessed using MTT assay after 24, 48, and 72 h. HCPT inhibited the growth of rabbits’ scleral fibroblasts in a concentration-dependent manner (Fig 1A). At HCPT concentrations above 2 μg/mL, a statistically significant decrease was noted in the survival rate of fibroblasts at 24 h compared with the control group (p < 0.05). At the HCPT concentration of 5 μg/mL for 48 h, the survival rate of fibroblasts was significantly different compared with the control group (p < 0.05) [19,20].

The morphology of rabbits’ scleral fibroblasts treated for 24 h with different HCPT concentrations was observed using inverted microscopy to understand the effect of HCPT on scleral fibroblast growth (S1 Fig). These observations indicate a significant inhibitory effect of HCPT on the growth of scleral fibroblasts in a concentration-dependent manner [21].

### HCPT induces G1 arrest of rabbits’ scleral fibroblasts

To determine whether the growth inhibitory effect of HCPT on rabbits’ scleral fibroblasts was associated with cell cycle arrest, scleral fibroblasts were treated with different HCPT concentrations for 24 h and analysed using flow cytometry. With increasing HCPT concentration, significant G0/G1 phase arrest was observed, indicating cell accumulation in the G1 phase (p < 0.05). HCPT concentrations of 2 and 5 μg/mL resulted in a significant decrease in the number of S phase cells (p < 0.05; Fig 1B and S2 Fig), similar to the results reported in a previous study [22].

### HCPT induces the apoptosis of rabbits’ scleral fibroblasts

To determine whether the inhibitory effect of HCPT on the proliferation of rabbits’ scleral fibroblasts was related to the induction of apoptosis, Annexin V and PI double staining was applied to detect apoptosis after treatment with various HCPT concentrations. HCPT significantly induced the apoptosis of scleral fibroblasts in a dose-dependent manner (Fig 1C and S3 Fig). The findings suggest that HCPT inhibits the growth of scleral fibroblasts by inducing their apoptosis [23,24].

### Application of HCPT HA hydrogel

**Fig 2:**
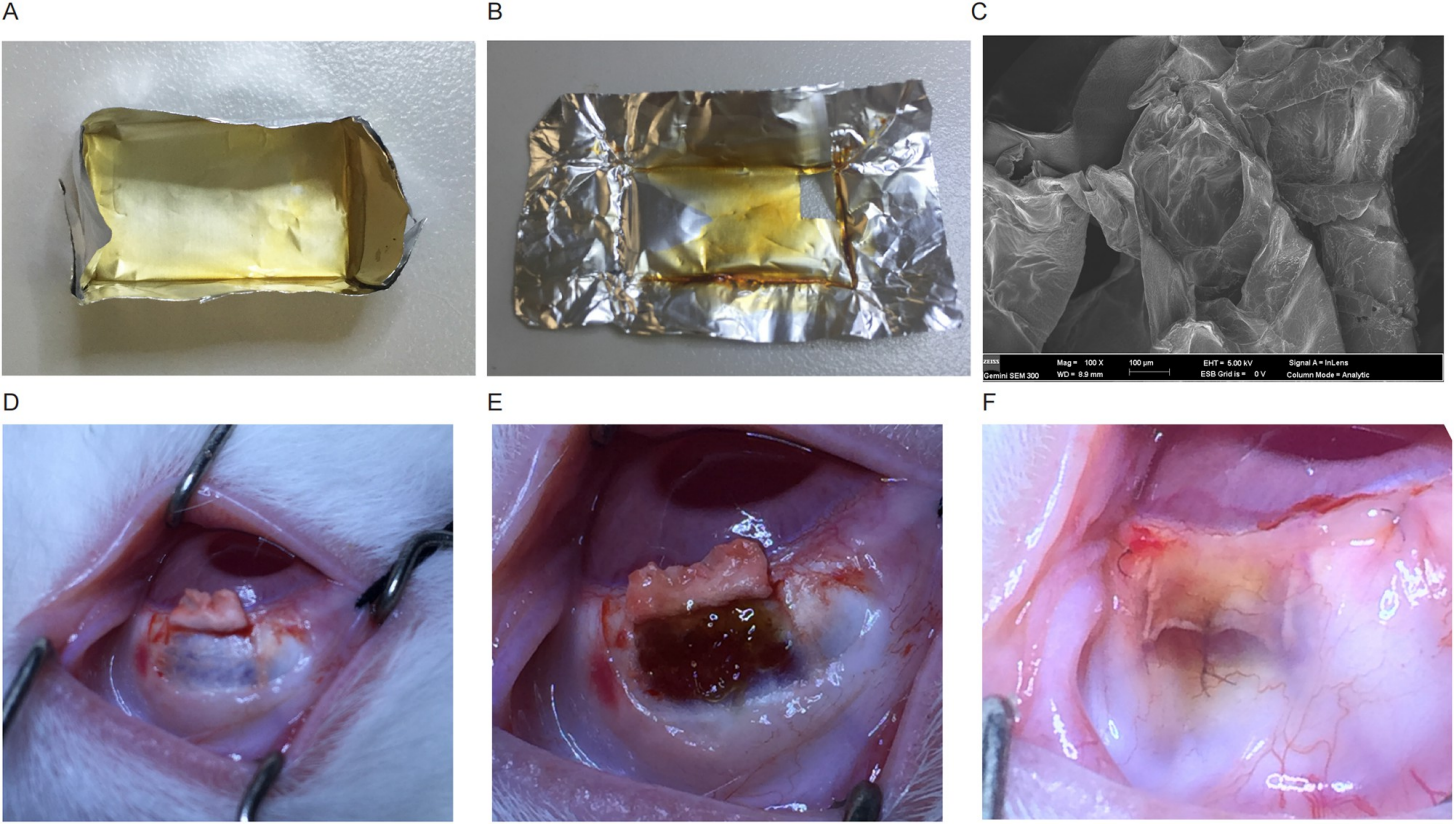
Preparation of HCPT hyaluronic acid (HA) hydrogel. (A) Preparation of HCPT-loaded HA hydrogels. (B) Dried films of 4×4 mm were placed under the scleral flap. (C) Representative electron micrograph of a hydrogel encapsulated with HCPT. (D-F) Three stitches were used to close the sclera. (E) Sutured conjunctiva.

Gradient HCPT (0.125, 0.25, and 0.5 mg/mL) was configured on HA hydrogel, and its microstructure was observed (Fig 2A–2C). Such pieces were placed under the scleral flap (Fig 2D and 2E), followed by three stitches to close the sclera (Fig 2F) and then the conjunctiva [25].

### Postoperative morphology of follicles and complications

**Fig 3:**
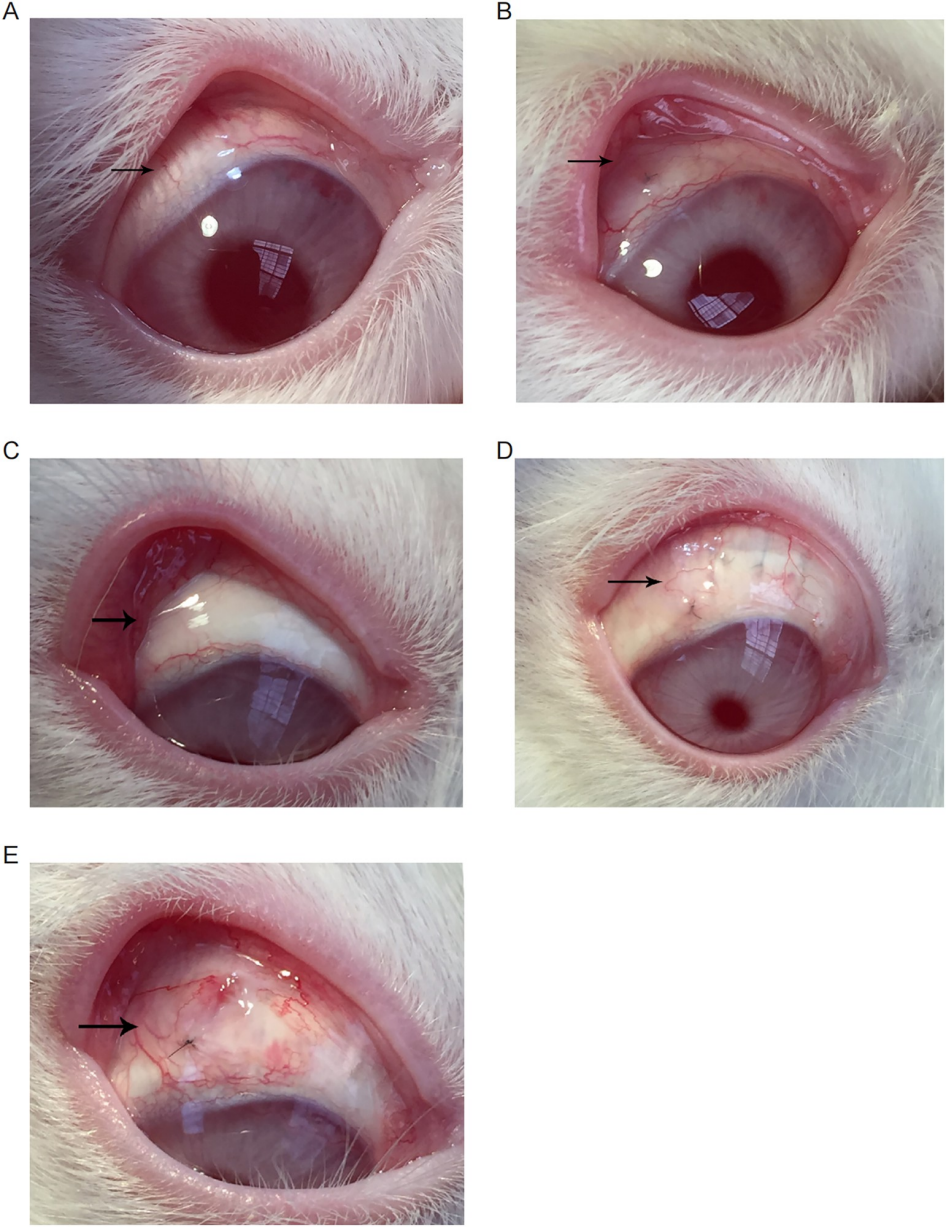
General observations after 1 week of treatment with HCPT hydrogel. One week after surgery, conjunctiva was marginally congested in each group, and the filter follicles were flattened in the control (A) and gel (B) groups. (C-E) Filter follicles were visible and elevated in the 0.125 mg/mL (C), 0.25 mg/mL (D), and 0.5 mg/mL (E) HCPT groups. The arrows in the figures show the filter bubbles.

**Fig 4:**
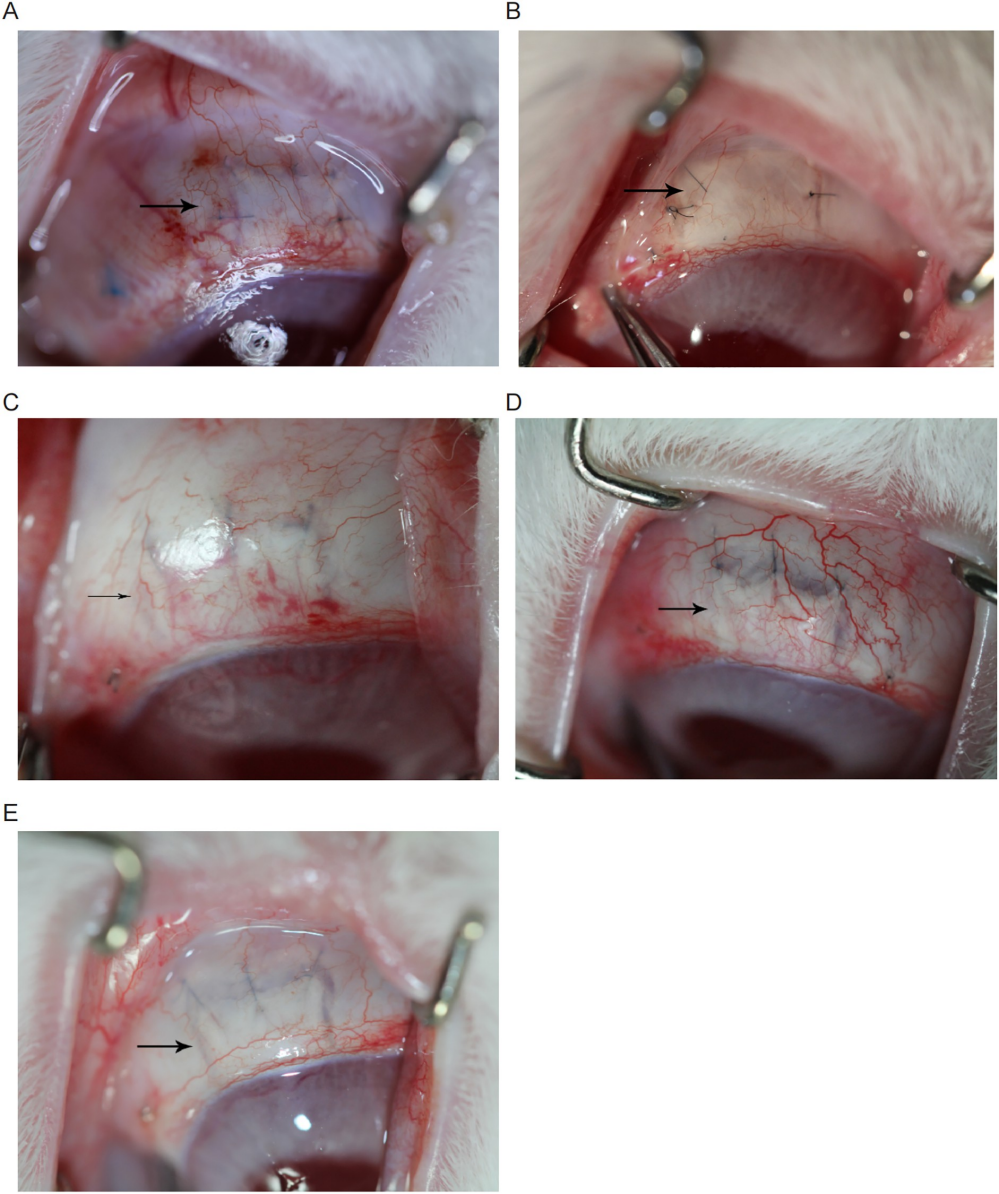
General observations after 2 weeks of treatment with HCPT hydrogel. Two weeks after surgery, conjunctivae of the control (A) and gel (B) groups were mildly congested. Filtration vesicles were absent, and the scleral laminae were well healed. (C-E) Conjunctivae of the HCPT group were not significantly congested, the filtration vesicles were diffuse and flattened, and the scleral laminae were visible and poorly healed. The arrows in the figures show the filter bubbles.

On the first postoperative day, the follicles in the five groups were clear, diffuse, and elevated. The follicle morphology in each group changed over time. One week after surgery, the conjunctiva was mildly congested in each group, and the follicles in the control (Fig 3A) and gel groups (Fig 3B) were flattened. Bulging was diffuse and elevated in the 0.125 mg/mL (Fig 3C), 0.25 mg/mL (Fig 3D), and 0.5 mg/mL (Fig 3E) HCPT groups. Two weeks after surgery, the conjunctiva in the control (Fig 4A) and gel (Fig 4B) groups was mildly congested without follicles. In the HCPT group (Fig 4C–4E), conjunctival hyperaemia was not evident, with diffuse flattening filtering bubbles [26].

Punctate exfoliation of the corneal epithelium was observed 3 days after surgery in the presence of various concentrations of HCPT, and healing was observed 1 week after surgery. One week after surgery, the follicles in the control group were flattened, whereas those in the other four groups were diffuse and elevated. Two weeks after the operation, filtration bubbles were not obvious in the control and gel groups but were found in the other three groups with diffuse and no thin-wall filtration bubbles. At 1 week postoperatively, small neovascularisation was observed at the local iris root in the trabeculectomy area, and at 2 weeks postoperatively, large neovascularisation was observed at the iris root (S4A–S4C Fig) [27].

### Effect of HCPT-loaded hydrogel on the gene expression of extracellular matrix protein in tissues

**Fig 5:**
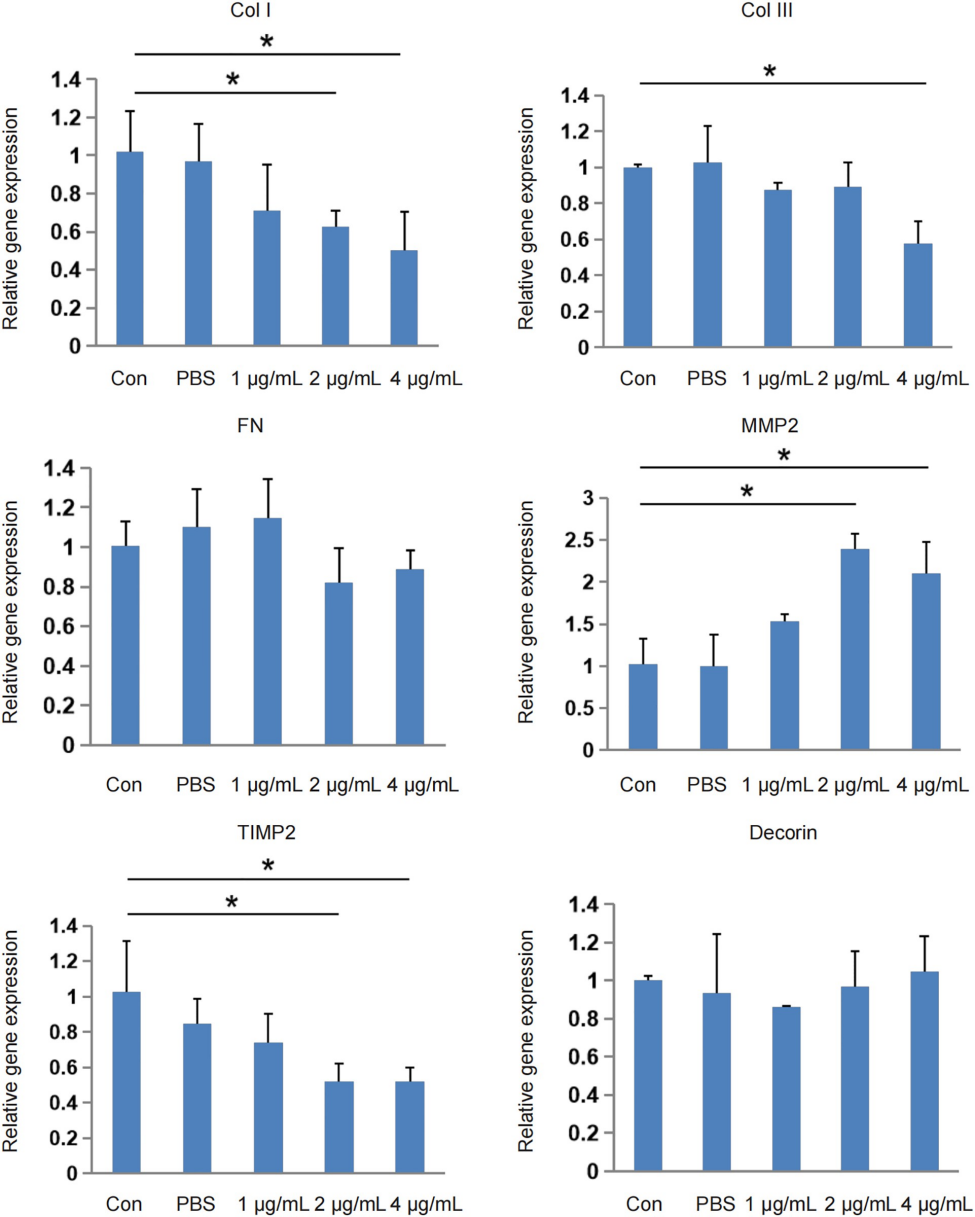
Effect of HCPT-loaded hydrogel on extracellular matrix protein gene expression. At high concentrations, HCPT hydrogels significantly inhibited the expression of collagen I (*COL I*), collagen III (*COL III*), and matrix metalloproteinase 2 inhibitor (*TIMP2*) genes in rabbit sclera tissues (p < 0.05). In contrast, matrix metalloproteinase 2 (*MMP2*) expression was significantly increased (p < 0.05). There were no significant effects on core proteoglycan (*Decorin*) and fibronectin (*FN*) gene expression.

When the HCPT concentration increased above 0.25 mg/mL, the expression of the scleral collagen 1 (*COL I*) gene was inhibited (p < 0.05). At 0.5 mg/mL HCPT, the expression of the scleral collagen 3 (*COL III*) gene was inhibited (p < 0.05). When the HCPT concentration exceeded 0.25 mg/mL, the expression of the scleral matrix metalloproteinase 2 (*MMP2*) gene was upregulated (p < 0.05) and correspondingly the expression of the tissue inhibitor of metalloproteinase 2 (*TIMP2*) gene was inhibited (p < 0.05). HCPT also inhibited the expression of the scleral fibronectin (*FN*) gene, but this was not statistically significant compared with the control group. HCPT had no significant effect on *decorin* gene expression (Fig 5).

### Effects of HCPT hydrogel on the expression of extracellular matrix protein in tissues

**Fig 6:**
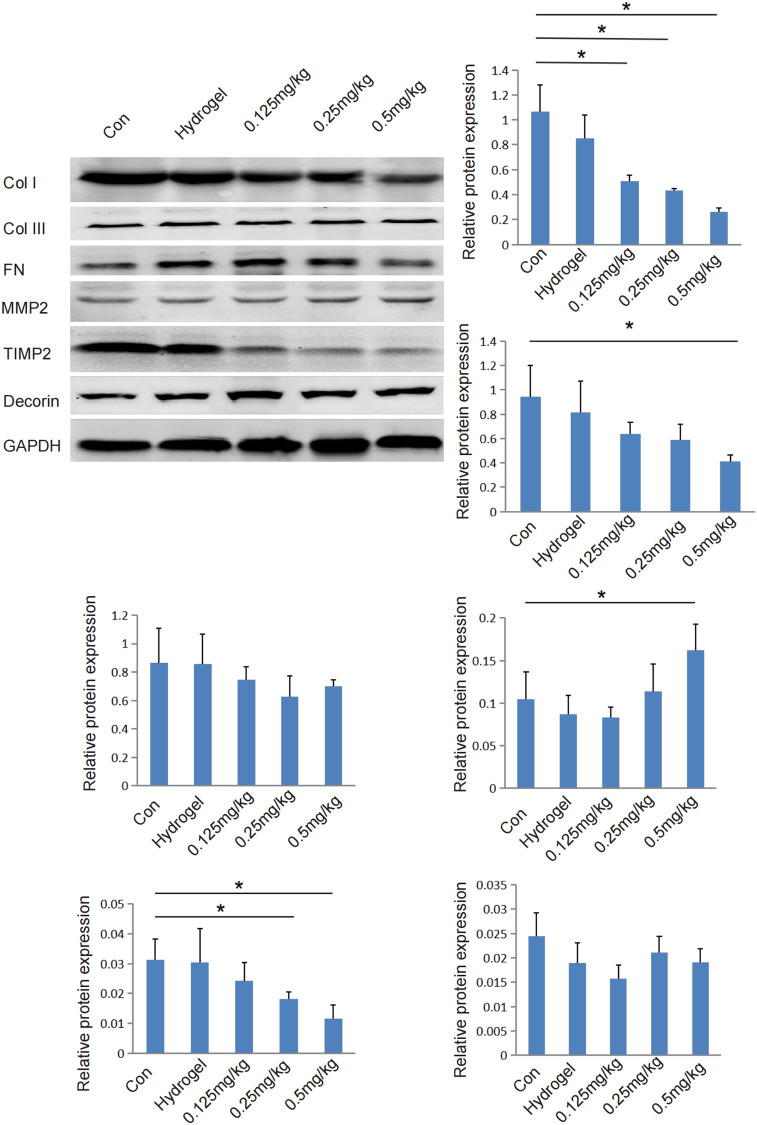
Effect of hydrogels containing different HCPT concentrations on the expression of extracellular matrix proteins in tissues. HCPT-loaded hydrogels (0.5 mg/mL) significantly inhibited the expression of COL I, COL III, and TIMP2 proteins in rabbit sclera tissue (p < 0.05). The expression of MMP2 protein was also significantly elevated (p < 0.05). No significant effects were observed on the expression of core proteoglycan (*Decorin*) and fibronectin (*FN*) proteins.

When the HCPT concentration exceeded 0.125 mg/mL, the expression of scleral COL I protein was inhibited (p < 0.05). When the HCPT concentration reached 0.5 mg/mL, the expression of scleral COL III protein was inhibited (p < 0.05). When the HCPT concentration exceeded 0.5 mg/mL, the expression of scleral MMP2 protein was upregulated (p < 0.05). When the HCPT concentration exceeded 0.25 mg/mL, the expression of scleral TIMP2 protein was prevented (p < 0.05). Similar to the gene expression results, the HCPT hydrogel had no significant effect on the expression of FN and decorin proteins (Fig 6) [28–30].

## Discussion

Postoperative wound healing and scar formation affect the success of glaucoma filtration surgery, which is an important obstacle to successful postoperative IOP control. This is mainly because tissue trauma caused by surgery causes the release of a variety of cytokines and inflammatory mediators, which stimulate the proliferation and migration of fibroblasts, leading to poorly functioning or even closed channels established by filtration surgery [31,32]. The wound healing process in filtration surgery is similar to the pathophysiological process after general surgery [33,34]. Therefore, some researchers believe that the 14 days following a glaucoma filtration surgery is a critical period for inhibiting scar formation. Researchers continue to improve filtration surgery and intraoperative medications to increase the success rate of surgery, which can reduce postoperative scar formation from the following aspects: First, reducing tissue damage in the surgical area can reduce the release of local inflammatory factors. Second, the topical use of steroid hormones or nonsteroidal anti-inflammatory eye drops before and after surgery can reduce inflammation. Third, antimetabolites, such as MMC and 5-FU, used during and after surgery can prevent excessive scar proliferation following glaucoma filtration. However, the toxic side effects of MMC and 5-FU restrict their applications [35,36].

HCPT is a derivative of camptothecin that has strong antitumor activity and low toxicity. HCPT has been used to treat various malignant tumours [37]. HCPT can inhibit DNA topoisomerase activity, leading to single-strand DNA breaks. As a cell cycle-specific drug, HPCT has no cross-resistance to common anti-tumour drugs. It can delay G2/M transition, mainly by acting on S phase cells. Owing to its anti-tissue fibrosis effect, HCPT is used to inhibit the proliferation of various fibroblasts, such as pathological scars, and to reduce the expression of collagen. In addition, the poor water solubility and serious side effects of HCPT limit its clinical application. Therefore, it is necessary to synthesise hydrogels with good solubility.

Few studies have addressed the use of HCPT in ophthalmology. HCPT can arrest rabbit lens epithelial cells in the S phase of the cell cycle and induce apoptosis and has a significant effect on inhibiting the proliferation of porcine retinal pigment epithelial cells. HCPT concentrations of 0.25–4.0 mg/L can effectively inhibit the proliferation of human Tenon’s fibroblasts [38]. HCPT also acts in the S phase of the cell cycle, stops cells in the G2 phase, has S phase cytotoxicity, and affects cell proliferation and activation. In addition, HCPT induces apoptosis in human conjunctival fibroblasts cultured in vitro and in primary cultured fibroblasts in subconjunctival scar tissue after anti-glaucoma surgery in a dose-dependent manner. Currently, the cannula filtration procedure for tube implantation and sclerostomy filtration can be used to establish animal models of glaucoma filtration duct scarring. The filtering method for tube implantation involves establishing a permanent subscleral channel by implanting a drainage tube, which facilitates the observation of filtering blebs after the operation. Conventional trabeculectomy was adopted as the sclerectomy filtering method. Postoperative inflammation and healing reactions are obvious, similar to the state of human glaucoma-filtering surgery. Therefore, we used the scarring model to filter conventional trabeculectomy.

We observed that HCPT inhibited hepatic stellate cell proliferation and the synthesis and secretion of COL type I in the extracellular matrix. In an in vitro study on glaucoma filtration using HCPT on filtration channel scar fibroblasts, HCPT significantly inhibited fibroblast proliferation and reduced collagen expression. In the present study, HCPT significantly inhibited the expression of type I and type III collagen genes and proteins in scleral fibroblasts. The inhibitory effects were enhanced with increasing drug concentrations. Our findings are similar to those of the aforementioned studies.

Although many studies have explored anti-scarring drugs after filtration surgery, only MMC and 5-FU have been used in clinical practice. These drugs have improved the surgical success rate to a certain extent. However, the use of MMC and 5-FU has been limited owing to side effects after their use, which can lead to serious complications. In this study, we provide the first evidence of the effect of HCPT on scleral fibroblasts and scar formation in the subscleral filtration channels. In a novel exploration, a hydroxycamptothecin hyaluronate hydrogel slow-release system was applied to rabbit eyes for filtration surgery. The effect was determined by observing COL I, COL III, MMP-2, TIMP-2, decorin, and FN protein gene and protein expression in the extracellular matrix and the localised expression of COL I and COL III to assess the anti-scarring effect of HCPT [39]. The combined present and previous results demonstrate that HCPT improves the success rate of glaucoma filtration surgery by inhibiting the proliferation of scleral fibroblasts cultured in vitro, reduces the synthesis of new collagen fibres in the scleral filtration tract after glaucoma filtration surgery in the early postoperative period, and inhibits scar formation in the scleral filtration tract. These results need to be confirmed in clinical randomised controlled trials.

## Conclusions

HCPT has a significant inhibitory effect on the growth of rabbits’ scleral fibroblasts, which increases in a concentration-dependent manner.

## Supporting information

S1 Fig Effect of hydroxycamptothecin on the growth of scleral fibroblasts in rats. Morphological observation of cells treated with different concentrations of HCPT was performed using inverted microscopy. With increasing HCPT concentrations, the number of scleral fibroblasts decreased significantly; the volume decreased; the cells were mostly round; the refractive property became poor; and the cells were suspended, detached, and died.
(TIF)

S2 Fig Effect of HCPT on the cell cycle of rat scleral fibroblasts. Cell cycle diagram of gradient HCPT drug therapy.
(TIF)

S3 Fig Effect of HCPT on the regulation of scleral fibroblasts in rats. Apoptotic diagram of gradient HCPT drug therapy.
(TIF)

S4 Fig Surgical complications. (A) At 1 week postoperatively, fine neovascularisation was visible at the local iris root in each group of trabeculectomy area. The arrows in the figures show normal iris blood vessels. (B-C) Large neovascularisation was visible at the local iris root in each group of trabeculectomy area. B. The arrows in the figures show small neovascularisation at the iris in the trabeculectomy area. C. The arrows in the figures show large neovascularisation at the iris in the trabeculectomy area.
(TIF)

S1 File (7Z)
