# Supplementary figures and images for: Hydroxycamptothecin regulates scar formation of the filtration channel under scleral flap by inhibiting the proliferation of scleral fibroblasts
Source: PLoS One. 2023 Apr 20;18(4):e0284618. doi: 10.1371/journal.pone.0284618 (PMC10118090; doi:10.1371/journal.pone.0284618)

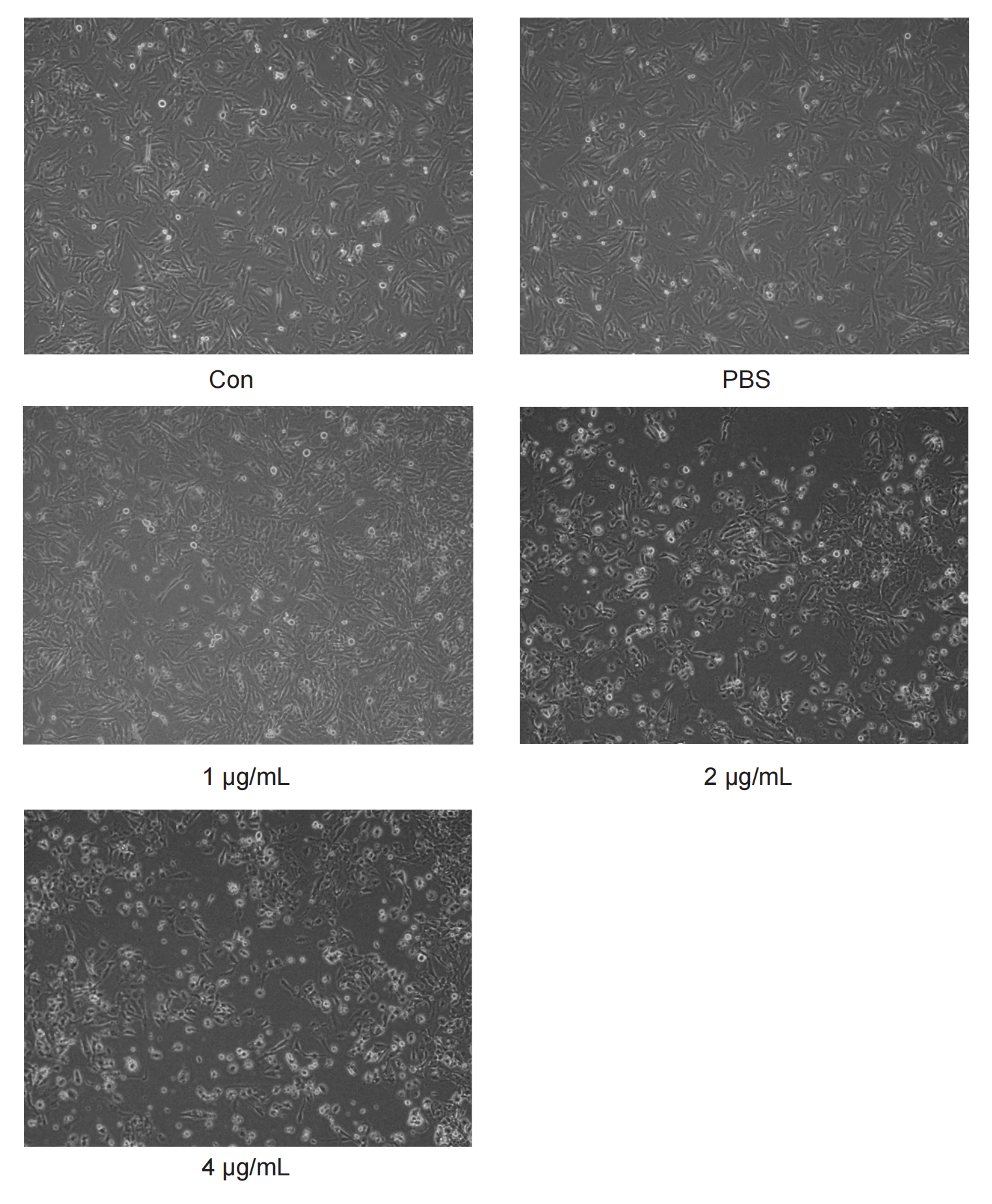

Supplement: S1 Fig — Morphological observation of cells treated with different concentrations of HCPT was performed using inverted microscopy. With increasing HCPT concentrations, the number of scleral fibroblasts decreased significantly; the volume decreased; the cells were mostly round; the refractive property became poor; and the cells were suspended, detached, and died. (TIF) [file pone.0284618.s001.tif]

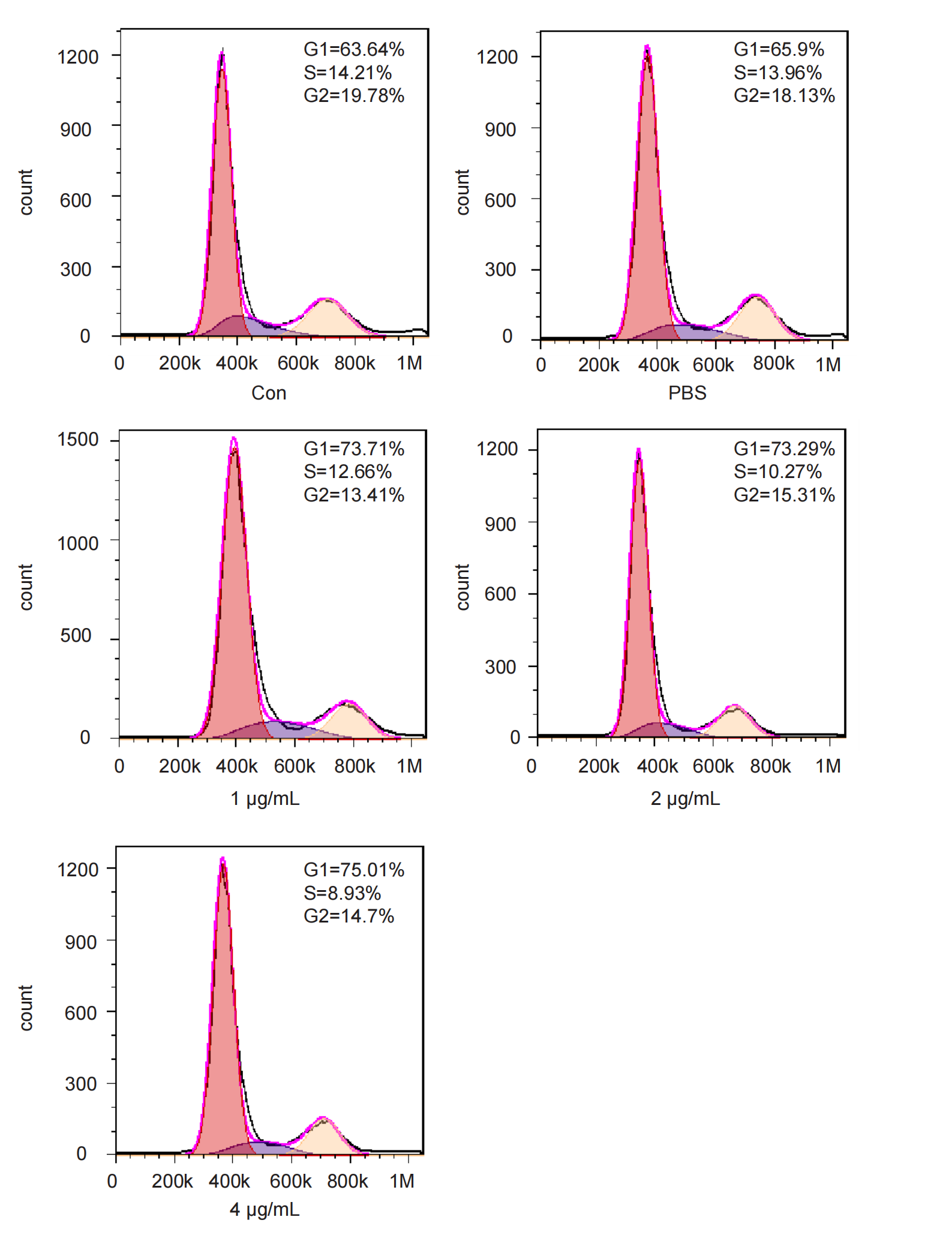

Supplement: S2 Fig — Cell cycle diagram of gradient HCPT drug therapy. (TIF) [file pone.0284618.s002.tif]

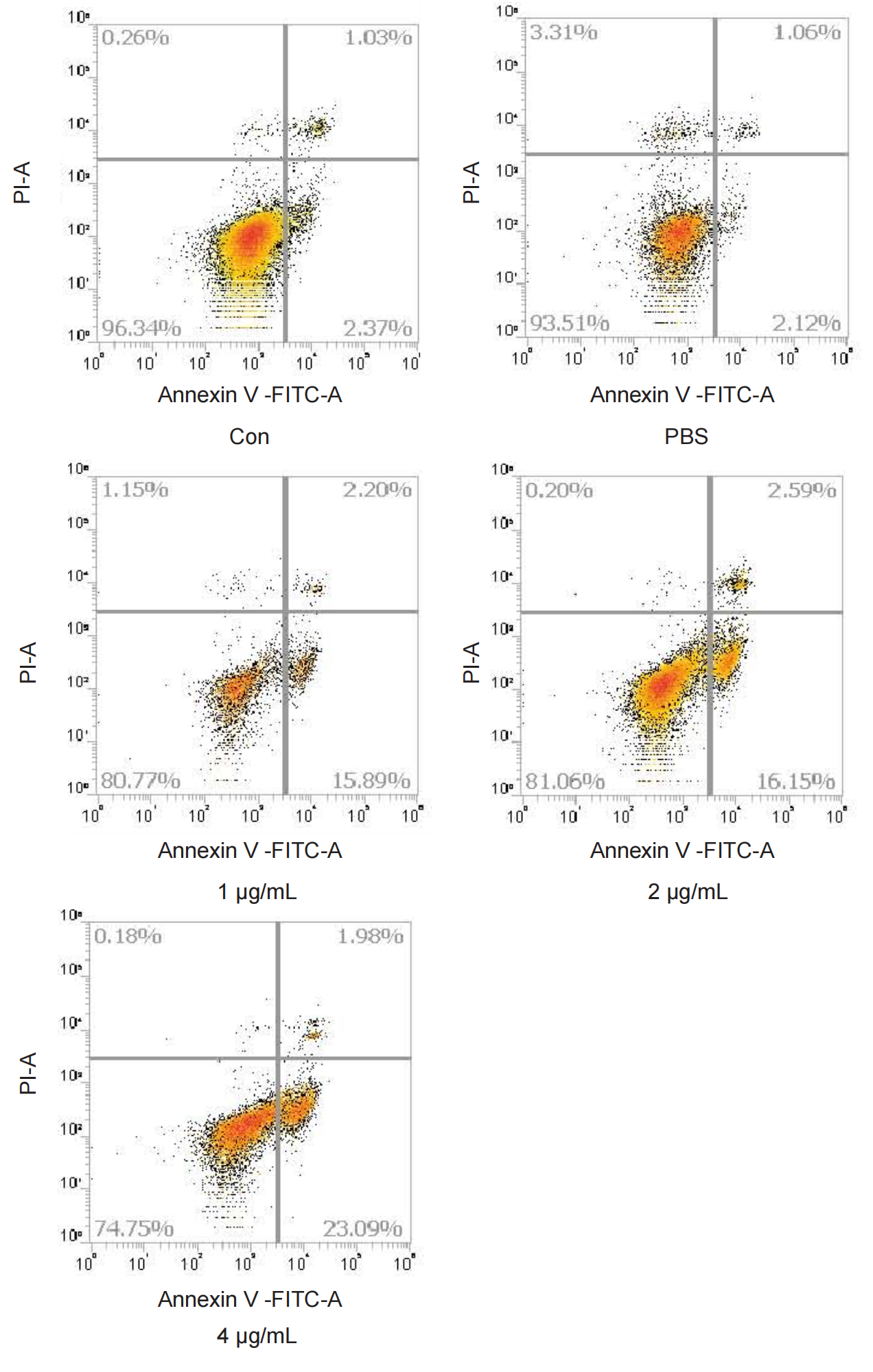

Supplement: S3 Fig — Apoptotic diagram of gradient HCPT drug therapy. (TIF) [file pone.0284618.s003.tif]

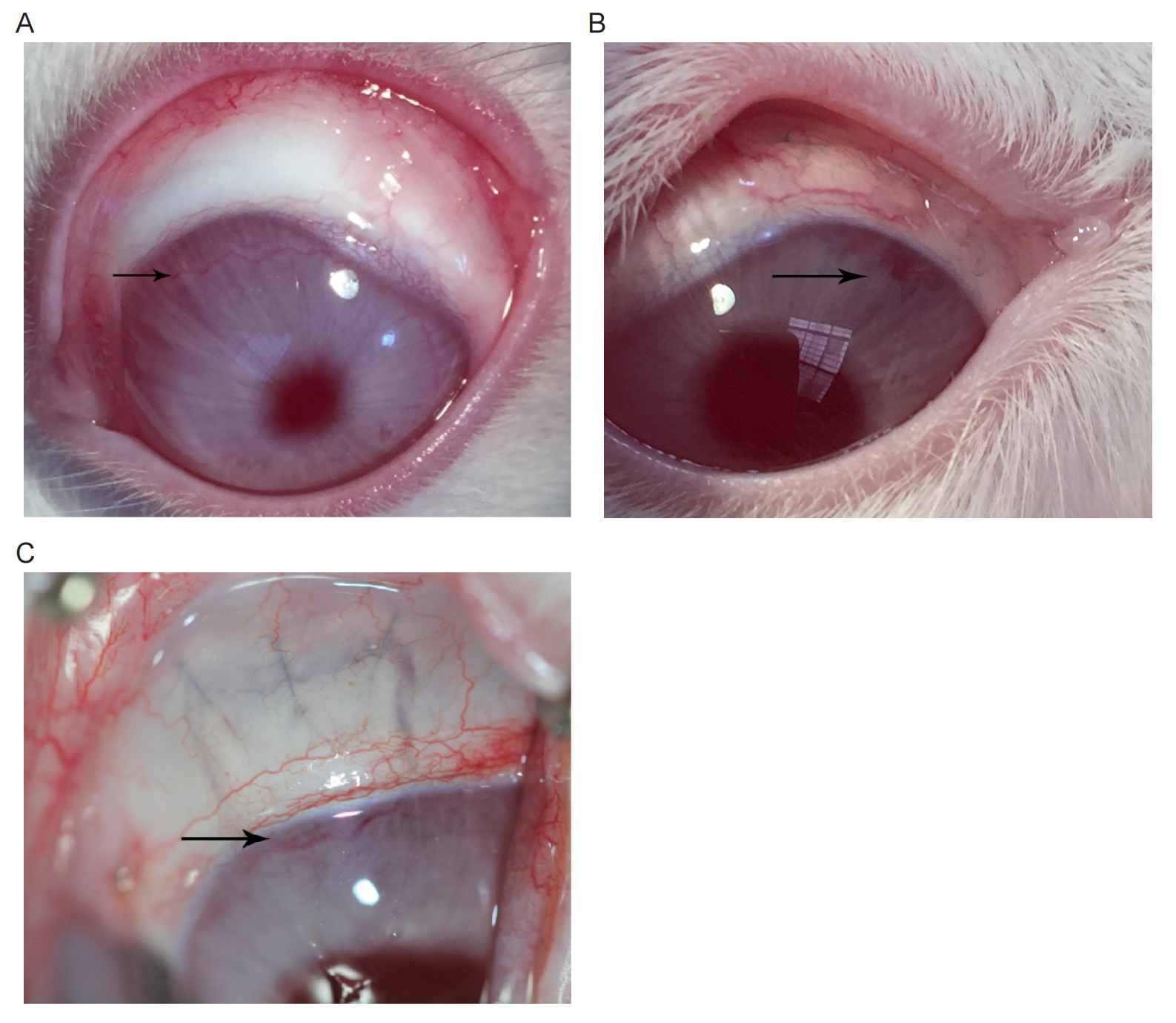

Supplement: S4 Fig — (A) At 1 week postoperatively, fine neovascularisation was visible at the local iris root in each group of trabeculectomy area. The arrows in the figures show normal iris blood vessels. (B-C) Large neovascularisation was visible at the local iris root in each group of trabeculectomy area. B. The arrows in the figures show small neovascularisation at the iris in the trabeculectomy area. C. The arrows in the figures show large neovascularisation at the iris in the trabeculectomy area. (TIF) [file pone.0284618.s004.tif]
